# Factors influencing PCV13 specific antibody response in Danish children starting in day care

**DOI:** 10.1038/s41598-020-63080-x

**Published:** 2020-04-10

**Authors:** Sine Fjeldhøj, Eva Fuglsang, Camilla Adler Sørensen, Hanne Frøkiær, Karen Angeliki Krogfelt, Rikke Pilmann Laursen, Hans-Christian Slotved

**Affiliations:** 1 Department of Bacteria, Parasites and Fungi, Statens Serum Institut, Copenhagen, Copenhagen 2300 Denmark; 2 Department of Veterinary and Animal Sciences, Faculty of Health and Medical Sciences, University of Copenhagen, Frederiksberg, 1870 Denmark; 3 Department of Science and Environment, Roskilde University, Roskilde, 4000 Denmark; 4 Department of Nutrition, Exercise and Sports, Faculty of Science, University of Copenhagen, Frederiksberg, 1958 Denmark

**Keywords:** Vaccines, Bacterial infection, Vaccines, Bacteriology

## Abstract

This study examines different factors influencing the 13-valent pneumococcal conjugate vaccine (PCV13) specific antibody response in 8–13 months old Danish children starting in day care. We present secondary findings to the ProbiComp study, which included nose swabs, buccal swabs and blood samples from the children before entering day care (baseline) and again after 6 months. Pneumococci isolated from nose swabs were identified by latex agglutination kit and Quellung reaction. Luminex-based assay was used for antibody measurements against specific anti-pneumococcal capsular IgG. Buccal gene expression was analyzed by qPCR. Statistical analyses were performed in R and included Pearson’s Chi-squared test, Welch two sample t-test and linear regression models. The PCV13 antibody response was unaffected by whether the children were carriers or non-carriers of any pneumococcal serotype. Having siblings increased the risk of carrying serotype 21 before day care (p = 0.020), and having siblings increased the PCV13 antibody response at the end of study (p = 0.0135). Hepatitis B-vaccination increased the PCV13 antibody response before day care attendance (p = 0.005). The expression of *IL8* and *IL1B* was higher in children carrying any pneumococcal serotype at baseline compared to non-carriers (p = 0.0125 and p = 0.0268 respectively).

## Introduction

Invasive pneumococcal disease (IPD) caused by *Streptococcus pneumoniae* (*S. pneumoniae*) is associated with high mortality and morbidity worldwide, especially among elderly aged 65+ years old and young children <2 years of age^1^. More than 92 different serotypes of *S. pneumoniae* are known^2^ and the 13-valent pneumococcal conjugate vaccine (PCV13), which was introduced into the Danish Childhood Immunization Program in 2010, protects against 13 of the known serotypes (serotype 1, 3, 4, 5, 6 A, 6B, 7 F, 9 V, 14, 18 C, 19 A, 19 F and 23 F)^1,3^. The use of effective pneumococcal conjugate vaccines in children led among all ages to a significant reduction in IPD caused by serotypes included in the vaccines, especially among children <2 years of age and elderly aged 65+ years old^1,4–7^. The PCV13 elicits antibody responses against the vaccine-included serotypes and thereby protects against vaccine serotypes^8^. Carriage of *S. pneumoniae* is most frequent in children <5 years of age, markedly in children <2 years of age, and pneumococcal carriage is a prerequisite for developing IPD^9,10^. Furthermore, a high carriage rate is associated with a high risk of respiratory infections^11^.

Children attending day care have an increased risk of pneumococcal carriage^10,12–14^ and a higher incidence of respiratory infections and hospitalizations due to acute respiratory infections compared to children in home care^15,16^. Around 90% of all Danish children aged one to two years old attend day care (Statistics Denmark, https://www.dst.dk/en, access 30-03-2020), hence it is important to examine strategies to reduce or prevent infections in children attending day care. The implementation of effective pneumococcal vaccines led to an increase in carriage of non-vaccine serotypes and an increase in the incidence of IPD caused by non-vaccine serotypes^1,3,5,10^. *S. pneumoniae* is known for capsular switching^17^. Thus, it is of great importance to continue the surveillance of pneumococcal carriage and to monitor the effect of pneumococcal vaccination on non-vaccine serotype carriage and subsequent development of diseases associated with *S. pneumoniae*.

The purpose of this study was to identify possible correlations between carriage of pneumococcal serotypes in PCV13-vaccinated children and the production of serotype-specific antibodies in children.

## Methods

### Design and study population

**Figure 1:**
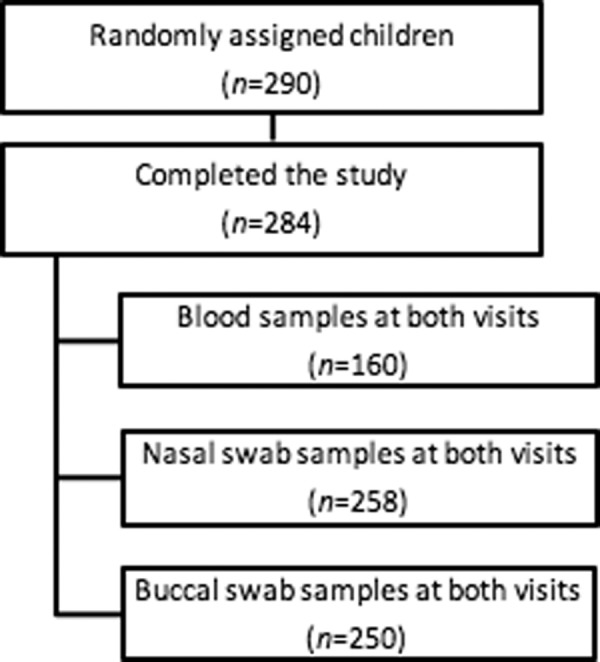
Flowchart of participants and obtained sample numbers per method.

This study presents secondary findings of the children included in the ProbiComp study^18^, which investigated the effect of a daily oral administration of a combination of the probiotics *Lactobacillus rhamnosus* GG (LGG) and *Bifidobacterium animalis subsp lactis* (BB-12) for six months to children aged 8–13 months at the time of day care enrolment. LGG and BB-12 are registered trademarks of Chr. Hansen A/S. The ProbiComp study was a randomized, double-blind, placebo-controlled parallel study which examined whether the probiotics influenced infections in young children and is described in details in Laursen *et al*.^18^. Figure 1 shows a modified flowchart of participants and obtained sample numbers per method as presented in Laursen *et al*.^18^.

Briefly, 290 healthy children aged 8–13 months starting day care within 12 weeks after start of intervention were included in the study. Recruitment was done over two seasons from mid-August to mid-December in 2014 and 2015. The children were clinically examined at two visits; one visit at baseline before starting in day care and one visit 6 months later at the end of study^18^.

At baseline and at the end of study nose and buccal swabs and a blood sample were drawn from the children and the carrier status and PCV13 geometric mean antibody response were analyzed by serotyping by a Luminex-based assay respectively.

Table 1 shows characteristics of study participants with nasal and oral swab samples from baseline and from the end of study. Data from the probiotic group and placebo group is combined as one group for this study. The table includes gender, siblings, breastfeeding prevalence, indoor pets, iron supplementation, health status since birth, age, vaccination (PCV13 and HepB coverage) and compliance (Table 1). Partial data in Table 1 has been presented in Laursen *et al*.^18^.

**Table 1:** Characteristics of study participants (*n* = 264).

Parameter	Total
**Probiotic group, n (%)**	132 (50.0)
**Female, n (%)**	129 (48.9)
**Siblings, n (%)**	134 (50.8)
**Breastfeeding prevalence:**	
1st visit, n (%)	127 (48.1)
2nd visit, n (%)	25 (9.5)
**Indoor pets, n (%)**	55 (20.8)
**Iron supplementation, n (%)**	95 (36.0)
**Health status since birth:**	
Doctor-diagnosed otitis media, n (%)	78 (29.5)
Doctor-diagnosed bronchitis, n (%)	23 (8.7)
Doctor-diagnosed pneumonia, n (%)	27 (10.2)
Oral/systemic antibiotic treatment	98 (37.1)
**Age:**	
1st visit (median months, IQR)	9.9 (9.5–10.4)
Start daycare (median months, IQR)	10.4 (9.9–11.2)
2nd visit (median months, IQR)	16.1 (15.6–16.6)
**Vaccination:**	
PCV-13 coverage ≤ 1 at visit 1, n (%)	264 (100.0)
HepB coverage ≤ 1 at visit 1, n (%)	122 (46.2)
HepB coverage ≤ 1 at visit 2, n (%)	136 (51.5)
**Compliance:**	
2nd visit (median % of consumed stics, IQR)	97 (94–99)

#### Vaccination data of the study population

Vaccination data were obtained at the Danish Vaccination Register (DDV) (record number 2015-57-0102). The vaccination data were used to assess the time between last given vaccination and blood sampling time. The data include information regarding which vaccines the specific child had received and date of vaccination. PCV13 is administered at the age of 3 months, 5 months and 12 months in a 2 + 1 dose schedule in Denmark^1^. At baseline, almost all the examined children (98.2%) were covered by at least one dose of PCV13, while 92.6% were covered by two doses. At the end of intervention 88.0% of the children had received all three doses of PCV13. Children with no vaccination data registered (n = 6) were excluded for analyses regarding vaccination status.

All children participating in the study have followed the Danish childhood vaccination program (except the aforementioned 6 children with no vaccination data registered). (https://en.ssi.dk/vaccination/the-danish-childhood-vaccination-programme, access 30-03-2020). In Denmark, hepatitis B vaccination is not included in the Danish childhood vaccination program but mainly recommended for people who are travelling outside of Denmark (https://en.ssi.dk/vaccination/travel-vaccination, access 30-03-2020). Almost half of the children were additionally covered by hepatitis B vaccination (137 children at baseline and 152 children at the end of study).

#### Nasal swab sampling

Nasal swab samples were collected by a modified version of Satzke *et al*.^19^ and were used to investigate carriage of *S. pneumoniae*. Briefly, minitip flocked swabs (FLOQSwabs^TM^, Copan, Italy) were inserted into the nasal cavity as far as possible but limited due to the child’s comfort. The swabs were placed in 1 mL Luria-Bertani (LB) broth with 10% glycerol in cryotubes and stored at −80 °C until analysis.

#### Identification of pneumococcal serotypes

The identification of pneumococcal serotypes was performed as previously described^9,10^ Briefly, inoculated serum-ox broth was incubated overnight at 37 °C in 5% CO_2_ before plating on 10% horse blood agar plates to increase sensitivity^9^. Pneumococcal serotypes were identified based on optochin sensitivity, bile solubility, α-hemolysis, pneumotest latex agglutination kit (SSI Diagnostica, Hillerød, Denmark) performed on the serum-broth and Quellung reaction (Neufeld test) using serotype specific antisera (SSI Diagnostica, Hillerød, Denmark). The specimens were screened by pneumotest latex agglutination kit for multiple serotypes. If multiple serotypes were found, they were isolated and serotyped.

### Blood sampling and sample preparation

The peripheral whole blood was drawn at baseline and at the end of intervention as previously described^8^. Aliquots (approximately 0.5 mL) were subsequently sampled according to the type of analysis and serum was collected and stored at −80 °C until examination by LuminexA. It was possible to collect blood samples from 252 children at baseline and 231 children at the end of study, however the analyses were limited by the aliquot volume of the collected blood, which was not always sufficient. The number of blood samples, nasal swabs and buccal swabs included for analyses is listed in flowchart 1.

### Antibody measurements of S. pneumoniae serotypes

The PCV13 geometric mean antibody responses were analyzed by a Luminex-based assay.

Luminex-based assay was used for antibody measurements against specific anti-pneumococcal capsular IgG (IgG-PN) as described by Kantsø *et al*.^20^. The assay was performed for 12 *S. pneumoniae* serotypes (1, 3, 4, 5, 6B, 7 F, 9 V, 14, 18 C, 19 A, 19 F and 23 F) since it was not possible to measure more than those serotypes with our current technology.

### Interleukin gene expression

Buccal gene expression was analyzed by qPCR. The collection of buccal swabs and measurement of gene expression by RT-qPCR was done as described in Hauger *et al*.^21^. In brief, the buccal swabs were collected by gently scraping the child’s inner cheek with a 0.6 mm interdental brush (TePe), which was immersed in isopropanol and lysis binding solution (MagMAX) after sampling and stored at −20 °C.

RNA was extracted with the MagMAX-96 Total RNA Isolation kit, and complementary DNA synthesized with the High Capacity with a total reaction volume of 10 μL consisting of 2 μL cDNA, 20 µL TaqMan gene expression assay and 2 µL TaqMan Fast Universal PCR Mastermix. cDNA Reverse Transcriptase kit (Applied Biosystems). Gene expression analysis was performed by qPCR on the StepOne instrument with universal fast thermal cycling parameters (ThermoFischer Scientific) on 2 μL cDNA, corresponding to 2.5 ng of RNA, in a total volume of 10 μL as previously described^21^.

Each sample was amplified in duplicates and the mean used in the analysis. The expression of interleukin *(IL)8* (*IL8*: Hs00174103_m1) and *IL1B* (IL1B: Hs01555410_m1 was measured, and Beta-actin (*ACTB*: Hs01060665_g1) was used as reference gene. Change in gene expression was calculated by the relative quantification cycle (Cq) method (2^-ΔΔCq^), where expression of each target gene was normalized to ACTB as ΔCq = Cq(target) - Cq(reference).

### Statistics

Baseline characteristics are shown as the mean (SD) or median (interquartile range [IQR]) for continuous variables and n (%) for categorical variables. Only children with nasal swabs, blood samples, or buccal swabs available for both visits were included in the analyses of carrier status, PCV13 vaccine response, or gene expression respectively. Differences according to having siblings, being vaccinated against hepatitis B, besides the Danish basic vaccination program, or being a nasal carrier or not were analyzed by linear regression models. For some analysis, a multivariate analysis was done to adjust for siblings as a potential confounder. All statistical analyses were performed in the software R (version 3.4.3; R Core Team, 2017. R: a language and environment for statistical computing. R Foundation for Statistical Computing, Vienna, Austria). An estimate was considered statistically significant at p < 0.05.

### Ethical considerations

The ProbiComp study protocol was approved by the Committees on Biomedical Research Ethics for the Capital Region of Denmark (H-4-2014-032). All methods were performed according to the guidelines and regulations approved by the Committees on Biomedical Research Ethics. Furthermore, the study was registered at clinicaltrials.gov (identifier NCT02180581, posted 02/07/2014)^18^. The study required informed consent from parents or other legal guardians of the children. Participation in the study was voluntary and parents could withdraw their consent at any time^18^.

## Results

Previous studies show no statistical significance between the probiotics group and the placebo group when comparing carriage rate of pneumococcal serotypes and no significant difference in antibody response to PCV13^8,10^. Furthermore, no significant differences between the groups were seen in terms of gender, siblings, breastfeeding, indoor pets, health status since birth, smoking in the household, age or compliance^18^, thus the two groups were compiled for analyses in this study (Table 1).

**Figure 2:**
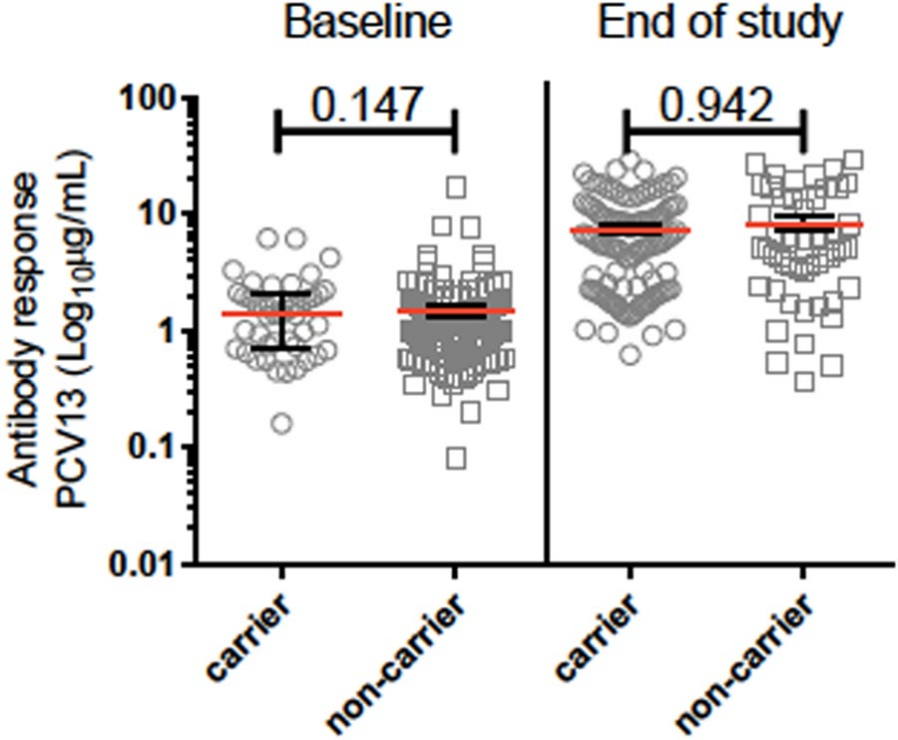
Antibody response to PCV13 and carriage of pneumococcal serotypes at baseline and at the end of study.

There was no statistically significant difference in antibody response to PCV13 vaccination when comparing carriers and non-carriers at baseline (p = 0.147) nor at the end of study (p = 0.942), (Fig. 2).

**Figure 3:**
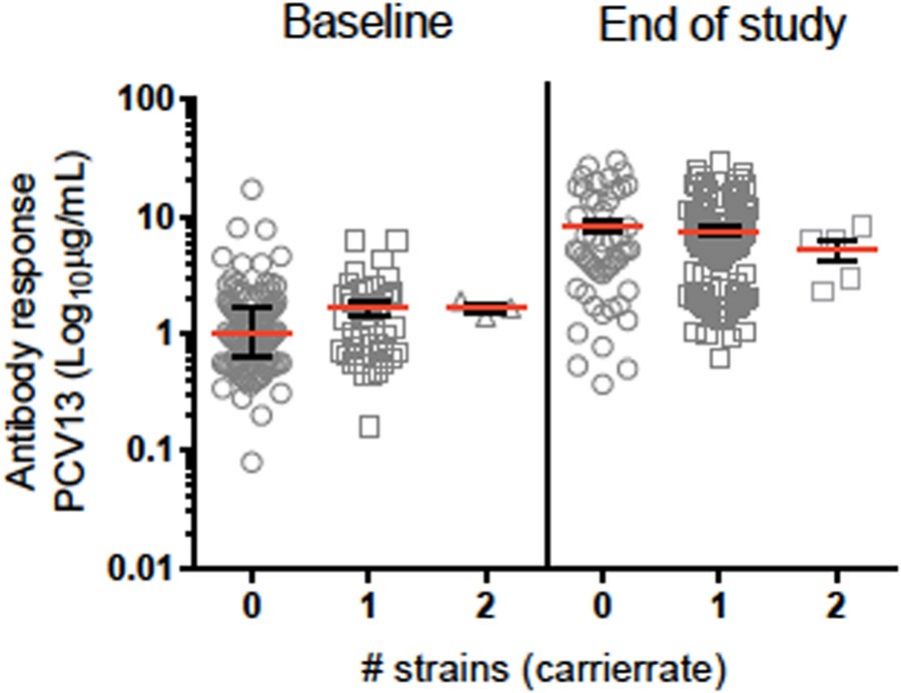
Antibody response to PCV13 and number of strains carried at baseline and at the end of study.

The PCV13 antibody response in children carrying 0, 1, or 2 pneumococcal strains was assessed. We found that the antibody response increased slightly with increasing number of strains carried at baseline, but tended to decrease with increasing number of strains carried at the end of study, yet neither the increase at baseline (p = 0.122) or the decrease at the end of study (p = 0.996) were considered significant (Fig. 3).

**Figure 4:**
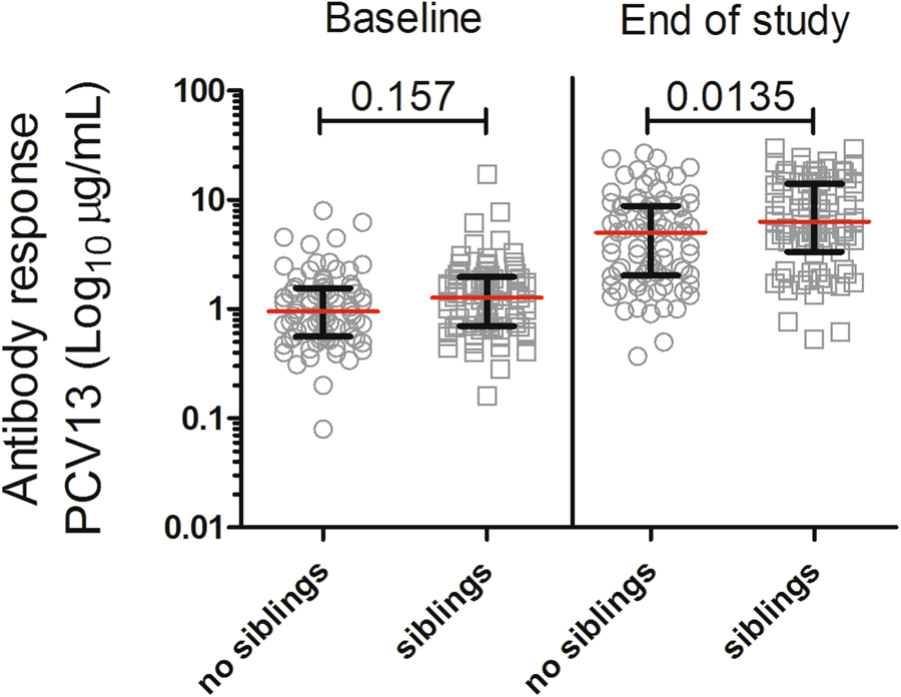
Antibody response to PCV13 (µg/mL) in children with or without siblings at baseline and end of study (no siblings, n = 81; siblings, n = 79). Having siblings increases the PCV13 antibody response at the end of study. At baseline and end of study, a blood sample was drawn from the children and PCV13 geometric mean antibody response were analyzed by a Luminex-based assay. Statistical analysis: Linear regression model, in R.

In a previous study^10^ we found that children living with siblings had a significant higher carriage rate of *S. pneumoniae* compared to only children. In this study, we assessed the PCV13 antibody response in children with and without siblings as novel information and found that at baseline there was no significant statistical difference in the PCV13 antibody response when compared children with and without siblings (p = 0.157), but having siblings increased the PCV13 antibody response (µg/mL) at the end of study (p = 0.0135) (Fig. 4).

**Figure 5:**
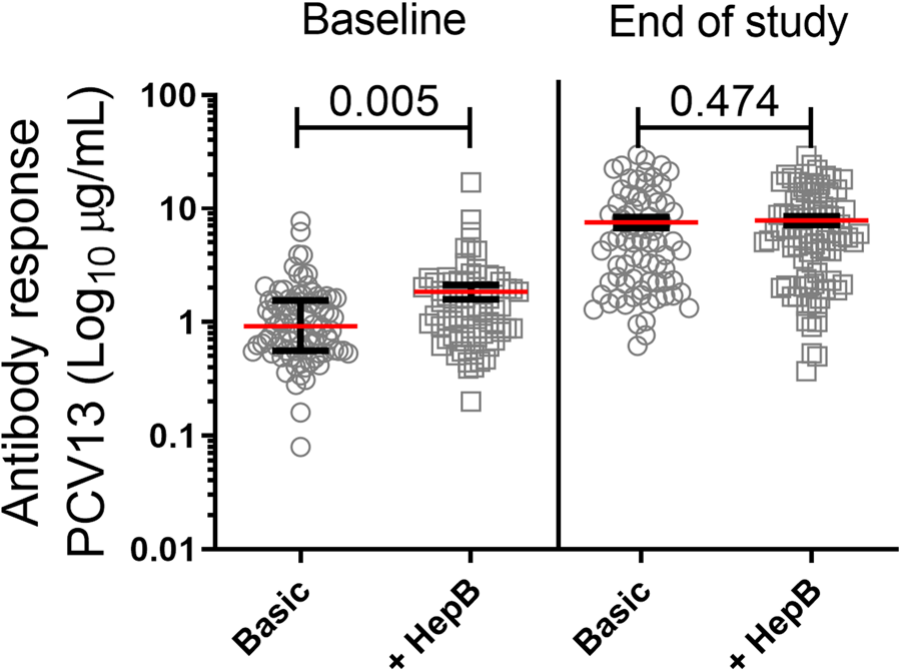
Being vaccinated against Hepatitis B increases the PCV13 antibody response at baseline but not at end of study in children that have followed the Danish basic vaccination program. At baseline and end of study, a blood sample was drawn from the children and the PCV13 geometric mean antibody responses were analyzed by a Luminex-based assay. The graph depicts the antibody response to PCV13 (Log_10_µg/mL) in children that have only followed the Danish basic vaccination program compared to children that along with the Danish basic vaccination program also have received Hepatitis B vaccination prior to either baseline or end of study (Baseline: Basic, n = 88; +HepB, n = 72; End of study: Basic, n = 75; +HepB, n = 85). Statistical analysis: Linear regression model in R.

The antibody response to PCV13 in children who received all vaccines included in the Danish childhood vaccination program and children who further received the hepatitis B-vaccination were compared in Fig. 5. Being vaccinated against hepatitis B in addition to the Danish childhood vaccination program increased the PCV13 antibody response at baseline (p = 0.005) but not at the end of study (p = 0.474) in children <2 years of age. Multivariate analysis adjusting for number of siblings showed that hepatitis B-vaccination significantly increased the PCV13 antibody response at baseline (p = 0.009) while the number of siblings had no impact on the PCV13 antibody response at baseline (p = 0.142). At the end of study hepatitis B-vaccination did not impact the PCV13 antibody response when adjusting for siblings (p = 0.468), but the number of siblings did have a significant impact (p = 0.019).

**Figure 6:**
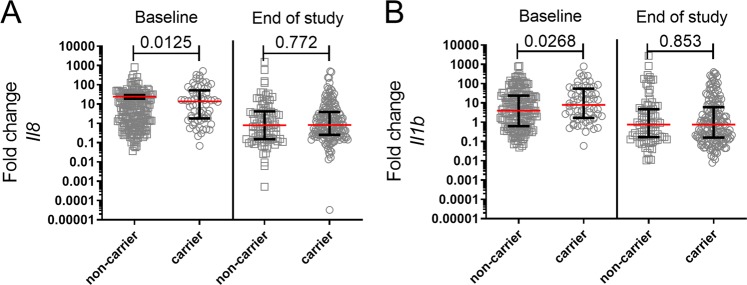
At baseline, but not end of study, oral mucosal gene expressions of IL-8 and IL-1β are increased in children that are carriers of any serotype in the nose. At baseline and end of study, a nose and a cheek swab were taken from the children and the carrier status in nose was analyzed by serotyping, while oral mucosal gene expression was analyzed by qPCR. Graphs depict the fold change of oral mucosal gene expressions of (**A**) IL-8 and (**B**) IL-1β at baseline and end of study in children that are either non-carriers or carriers of any serotype in the nose (Baseline: non-carrier, n = 190; carrier, n = 64. End of study: non-carrier, n = 83; carrier, n = 172), Statistical analysis: Linear regression model in R.

Buccal gene expressions of *IL8* and *IL1B* in children that were carriers of pneumococcal serotypes and non-carriers were tested (Fig. 6). The expression of *IL-8* and *IL-1B* was higher in children who carried any serotype in the nose at baseline compared to non-carriers, but not at the end of study. Multivariate analysis was performed to examine whether the number siblings impacted the correlation between buccal gene expression of *IL8* and *IL1B* and carrier status of pneumococcal serotypes. Being a carrier of *S. pneumoniae* significantly increased the gene expression of *IL8* (p = 0.044) and *IL1B* (p = 0.038) at baseline when adjusting for number of siblings. Though at the end of study neither the number of siblings (p = 0.995) nor being a carrier of *S. pneumoniae* (p = 0.772) had an impact on *IL8* expression or *IL1B* expression (p = 0.778 and p = 0.848 for siblings and carrier status, respectively), when performing multivariate analysis.

The antibody response to specific serotypes depending on the carriage of specific serotypes is included as a supplementary figure. E.g. the antibody response to serotype 19 F was lower in children carrying serotype 23B compared to children not carrying serotype 23B (p = 0.0453), although the number of children carrying 23B was small (four carriers compared to 156 non-carriers) making the results questionable. For the majority of the serotypes it was not possible to calculate differences in antibody responses in children carrying specific serotypes or not due to low numbers of children being carriers compared to non-carriers (supplementary figure 1).

## Discussion

As a secondary finding of the ProbiComp study, we have assessed the association between the PCV13 antibody response and the carriage status of *S. pneumoniae* serotypes in healthy children aged 8–13 months starting in day care. It was previously shown that daily administration of a combination of *Bifidobacterium animalis* ssp. *lactis* and *Lactobacillus rhamnosus* GG for a 6-month intervention period did not affect the antibody response against *S. pneumoniae* in healthy Danish children in terms of geometric mean of the antibody concentrations against 12 *S. pneumoniae* serotypes^8^. In this study, we found that the antibody response to PCV13-vaccination was independent of the pneumococcal carrier status of the children.

Carrying a pneumococcal serotype is a prerequisite for developing IPD^9^. A study by Kimaro Mlacha *et al*. found that individuals respond with different dynamics following stimulation with pneumococcal polysaccharide antigens, and children aged 24–36 months with a history of IPD responded quicker than the healthy controls^22^. This may be due to a memory response elicited by the serotype that caused IPD^23^ or because the children may have been carrying pneumococcal serotypes beforehand. A study by Ingels *et al*. also showed the importance of evaluating pneumococcal antibody response on an individual level in children at higher risk of IPD^24^. The reason why we did not see a difference in PCV13 antibody response between carriers and non-carriers in our study may have several causes. Firstly, there might be individual differences in terms of responding time following PCV13 vaccination. Secondly, the antibody responses might have been shaped by prior natural exposure to pneumococcal antigens in nasopharyngeal carriage. Children who are exposed to pneumococci may be more likely to have acquired a wide repertoire of memory responses to different serotypes as proposed by Kimaro Mlacha *et al*.^22^. Thirdly, the antibody response to PCV13-included serotypes might not be affected by the antibody response to a non-vaccine serotype. However, we did see that the antibody response to some serotypes was affected by the carriage of other serotypes (supplementary figure 1), e.g. the antibody response to serotype 1 or 18 C were both higher in children carrying non-vaccine serotype 23 A than in children not carrying 23 A at baseline (before entering day care), and the antibody response to 18 C was also higher in children carrying serotype 11 A compared to children not carrying serotype 11 A at baseline. However, these analyses included few carriers of specific serotypes compared to non-carriers of that serotype and are therefore not reliable. Yet it cannot be excluded that colonization by one serotype may protect against colonization by another serotype, as competition-colonization between some pneumococcal serotypes have been reported^25^.

Our previous study^10^ showed a statistically significant difference between non-carriers and carriers in terms of living with or without siblings at baseline, showing a higher risk of carrying pneumococcal serotypes before day care attendance if the child has at least one older sibling. This study on the other hand shows that the PCV13 antibody response was not affected by the number of siblings at baseline, but a higher PCV13 antibody response was seen in children with siblings at the end of study. Children at the end of the study received an extra dose of PCV13 compared to children at baseline. The number of siblings may affect the carriage status before day care attendance but not the PCV13 antibody response.

We found that vaccination against hepatitis B significantly increased the PCV13 antibody response at baseline (p = 0.009) but not at the end of study (p = 0.468) in children who followed the Danish childhood vaccination program when adjusting for number of siblings as a potential risk factor for pneumococcal carriage. The non-significant finding at the end of the study might be because of day care attendance throughout 6 months, where the children were affected by several other external environmental factors such as more vaccinations and other microorganisms that cannot be accounted for in this study. However, early in life and prior to day care, the hepatitis B vaccine might affect the protection against pneumococcal serotypes by a bystander non-specific immune stimulation leading to an increase in the PCV13 antibody response. To our knowledge, the association between the PCV13 antibody response and the hepatitis B vaccine has not been described in other studies, but it could be considered whether to exploit this additive effect on PCV13 response along with additional hepatitis B protection in the Danish childhood vaccination program.

Our study shows an increase in the buccal mucosal gene expressions of *IL8* and *IL1B* in children that are carriers of any pneumococcal serotype in the nasal cavity at baseline even when adjusting for number of siblings as a potential risk factor for carriage of *S. pneumoniae*. Other studies have found a higher expression of *IL8* and *IL1B* in serum in hepatitis B virus (HBV) infected patients^26–28^, while another study found mainly IL-10 to be expressed in HBV infected patients and HBV-vaccinated individuals^29^. Mucosal expression of *IL8* and/or *IL1B* indicates activation of the mucosal immune system, which is expected in the presence of bacteria^30^. We measured the expression of the cytokines in buccal swabs, while the carriage of pneumococci was determined from nose swabs. It is however expected that microorganisms found in the nose will be present in the mouth and throat as well, and vice versa. *IL8* and *IL1B* play a crucial role in protection against both hepatitis B and *S. pneumoniae*, and our findings of an increase in PCV13 antibody response in children vaccinated against hepatitis B might be due to of an increased activation of the mucosal immune system. Our findings of an increased expression of *IL8* and *IL1B* in pneumococcal carriers is supported by others^30–32^. Neutrophils are known to be critical in the protection against and the clearance of *S. pneumoniae*^33,34^ and are recruited to the site of colonization through IL8. Morris *et al*. (2017) found that the *S. pneumoniae* burden was significantly correlated with neutrophil recruitment and transcription of *IL8* and that the transcription of *IL8* in neutrophils was correlated with the secretion of IL8 into the nasopharynx^30^, while Marriott *et al*. (2012) showed that IL1B regulates IL8 release from epithelial cells in response to *S. pneumoniae*^32^. This might explain our finding of an increased gene expression of *IL8* and *IL1B* in children carrying pneumococcal serotypes, however the oral cavity is highly polymicrobial and *IL8* levels can be upregulated in response to the presence of other bacteria than *S. pneumoniae* as well. We see a correlation between buccal gene expressions of *IL8* and *IL1B* and carriage of pneumococcal serotypes, yet we cannot exclude that other microorganisms affect this correlation.

In conclusion, the PCV13 antibody response was unaffected by whether the children were carriers or non-carriers of any pneumococcal serotype and entering day care equalized most effects of *S. pneumoniae* colonization measured before entering the day care. Children <2 years of age living with older siblings were more frequent carriers of pneumococcal serotypes before day care attendance, especially of the non-vaccine serotypes 21 and 11 A, compared to children not living with siblings. Vaccination against hepatitis B increased the PCV13 antibody response in children who followed the Danish childhood vaccination program before they entered day care. Lastly, we saw an increased mucosal gene expression of *IL8* and *IL1B* in children carrying pneumococcal serotypes before day care attendance.

In the future, it is important to continue the surveillance of pneumococcal carriage and to monitor the effect of pneumococcal vaccination on carriage of non-vaccine serotypes. Further research is needed to investigate whether hepatitis B vaccination protects against pneumococcal carriage by increasing the PCV13 antibody response or by an increased gene expression of *IL8* and *IL1B*. Lastly, further investigations are needed to examine whether the antibody response to one specific serotype is depended on the carriage of another serotype.

## Supplementary information

Supplementary figure.
