# Supplementary material for: Factors influencing PCV13 specific antibody response in Danish children starting in day care
Source: Sci Rep. 2020 Apr 10;10:6179. doi: 10.1038/s41598-020-63080-x (PMC7148338; doi:10.1038/s41598-020-63080-x)
Supplement: Supplementary file 1 — Supplementary figure. [file 41598_2020_63080_MOESM1_ESM.docx]

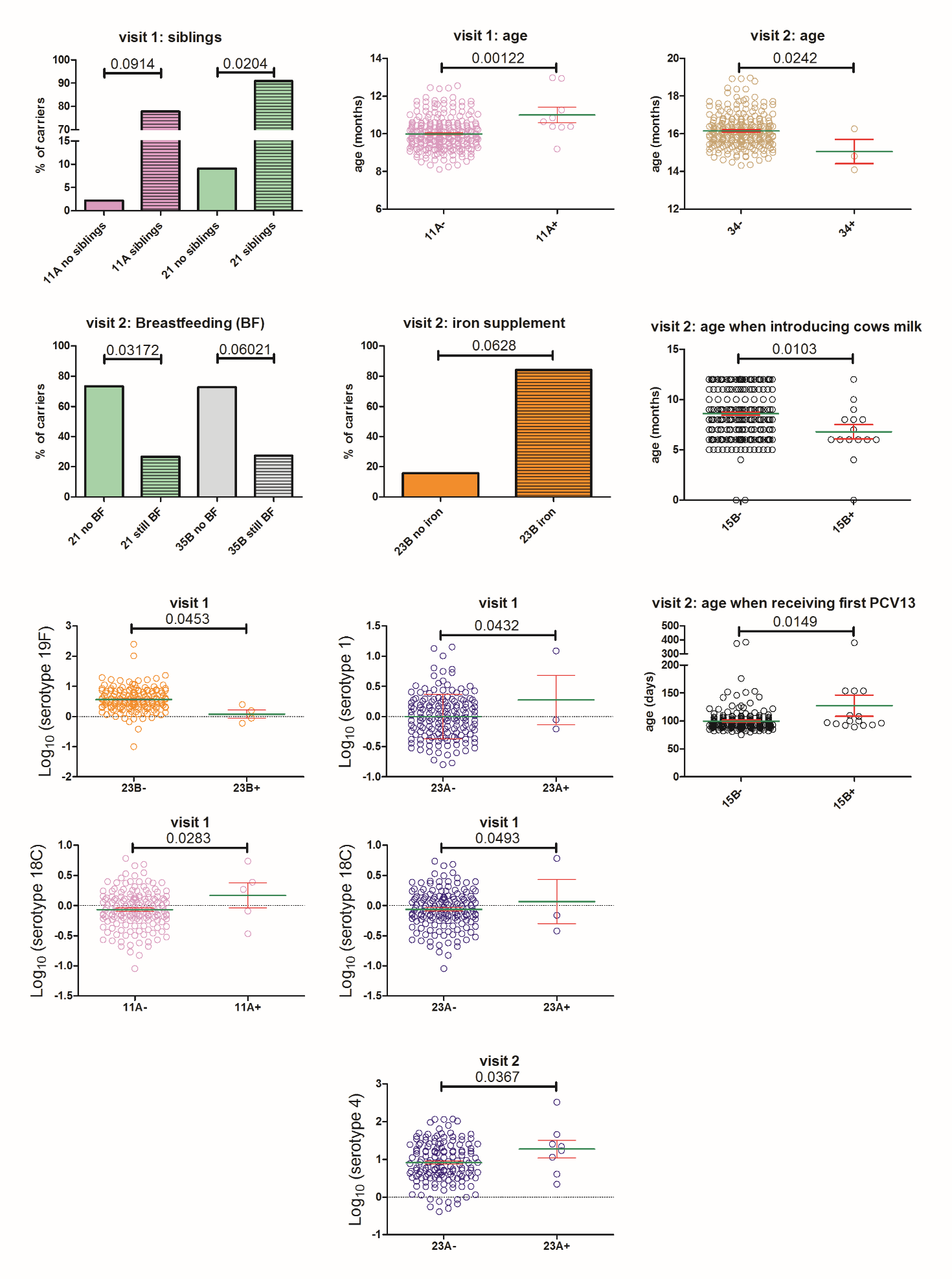


Supplementary figure 1: Antibody response to specific serotypes depending on carriage of specific serotypes. Visit 1: Antibody response to serotype 18C depending on carriage of 11A (11A-, n=155; 11A+, n=4), antibody response to serotype 1 depending on carriage of 23A (23A-, n=157; 23A+, n=3), antibody response to serotype 18C depending on carriage of 23A (23A-, n=157; 23A+, n=3), antibody response to serotype 19F depending on carriage of 23B (23B-, n=156; 23B+, n=4). Visit 2: Antibody response to serotype 4 depending on carriage of 23A (23A-, n=151; 23A+, n=8). Statistics: univariate logistic regression.
